# Macrophage accumulation in dorsal root ganglion is associated with neuropathic pain in experimental autoimmune neuritis

**DOI:** 10.1515/tnsci-2022-0355

**Published:** 2024-10-24

**Authors:** Chunrong Li, Fangzheng Cao, Houwen Zhang, Weijiao Fan, Yifan Cheng, Yao Lou, Yiqi Wang

**Affiliations:** Center for rehabilitation Medicine (Affiliated People’s Hospital, Hangzhou Medical College), Hangzhou, China; The Second Clinical Medical College of Zhejiang Chinese Medical University, Hangzhou, China; Clinical Research Institute, Key Laboratory of Tumor Molecular Diagnosis and Individualized Medicine of Zhejiang Province, Zhejiang Provincial People’s Hospital (Affiliated People’s Hospital, Hangzhou Medical College), Hangzhou, China; Hangzhou Medical College, Hangzhou, China; School of Basic Medical Sciences and Forensic Medicine, Hangzhou Medical College, Hangzhou, Zhejiang, China

**Keywords:** macrophages, dorsal root ganglion, neuropathic pain, experimental autoimmune neuritis

## Abstract

**Background:**

Neuropathic pain is a common symptom of Guillain–Barré syndrome (GBS). The infiltration of macrophages in the dorsal root ganglion (DRG) contributed to neuropathic pain in nerve injury. The underlying mechanisms of neuropathic pain in patients with GBS remain unknown. Experimental autoimmune neuritis (EAN) is a useful mice model of GBS. Our study aimed to explore whether the infiltration of macrophages in DRG is associated with neuropathic pain of EAN.

**Methods:**

Male C57BL/6 mice were randomly divided into two groups, the EAN group (*n* = 12) and the control group (*n* = 12). Six mice in each group were sacrificed after anesthetization in the attack and remission phase, respectively. The 50% paw withdrawal threshold and clinical score were measured, and macrophages with its subtypes were detected in the spleen and DRG tissue.

**Results:**

More macrophages infiltrated the DRG of the EAN group in the attack phase and mostly surrounded neurons in the DRG. The proportion of macrophages and pro-inflammatory macrophages in the spleen of mice with EAN was significantly higher than the control group in the attack phase.

**Conclusion:**

The infiltration of macrophages in DRG might be associated with neuropathic pain of EAN and pro-inflammatory macrophages may involve in neuropathic pain of EAN.

## Abbreviations

GBS: Guillain–Barré syndrome
PNS: peripheral nervous system
DRG: dorsal root ganglion
EAN: experimental autoimmune neuritis
FCA: Freund’s complete adjuvant
PBS: phosphate-buffered saline
MNCs: mononuclear cells
Arg-1: anti-mouse arginase-1
iNOS: inducible nitric oxide synthase
HMGBQ: high mobility group box 1

## 1 Introduction

Guillain–Barré syndrome (GBS) is a common autoimmune disease characterized by inflammatory infiltration and damage to myelin and axon in the peripheral nervous system (PNS). Common clinical manifestations include areflexic paresis and sensory abnormalities. Neuropathic pain is observed in 55–85% of GBS patients, which is often overlooked due to the rapid progression of paresis [1]. However, the underlying mechanisms responsible for neuropathic pain in GBS remain unknown. Dorsal root ganglion (DRG), a critical structure involved in sensory transduction and modulation, has been implicated in the development and maintenance of neuropathic pain [2]. Recent studies have demonstrated that accumulation of macrophages in DRG can induce neuropathic pain both in animal models of nerve injury and peripheral neuropathy caused by paclitaxel chemotherapy [3,4].

Experimental autoimmune neuritis (EAN) is an immune-mediated inflammatory disease of the PNS and serves as a valuable animal model of GBS [5]. Increasing evidence suggests that macrophages play significant roles in GBS [6,7,8]. Macrophages are the main effector cells in the pathogenesis of EAN [9]. Directly phagocytosing myelin and releasing pro-inflammatory cytokines as well as noxious molecules, contributing to immune-mediated nerve damage of EAN [9]. The alteration of the balance of pro-inflammatory macrophages/anti-inflammatory macrophages has been shown to attenuate clinical severity in EAN [6,7,8]. Previous studies on EAN have primarily focused on the clinical scores measured by motor deficits [7,10]. Neuropathic pain has also been reported in EAN by several studies [11]. Xu et al. found that oridonin, a natural diterpenoid could suppress neurological progression, Mechanical Allodynia, and the number of accumulated macrophages in sciatic nerves in EAN [6]. However, there have been no studies investigating whether macrophage infiltration in the DRG leading to neuropathic pain in EAN.

In the present study, our hypothesis was that there is infiltration of macrophages in the DRG, which is associated with mechanical allodynia in mice with EAN. Furthermore, we propose that the presence of pro-inflammatory macrophages may be associated with hyperesthesia in EAN.

## 2 Methods

### 2.1 Animals and reagents

Male C57BL/6 mice (6–8 weeks, weight 16–18 g) were obtained from Slack Experimental Animal Co., Ltd. (Shanghai, China) and acclimated to the animal feeding room of Zhejiang University of Technology for 1 week before the experiments. The room temperature was maintained between 21 and 25℃. Regular feeding and free water were provided. The experiment was approved by the ethics committee of Zhejiang Provincial People’s Hospital (SYXK2018-0012). The mice were randomly divided into two groups, the EAN group (*n* = 12) and the control group (*n* = 12); six mice in each group were sacrificed after anesthetization in the attack (day 14 post-immunization [p.i.]) and remission phase (day 32 p.i.), respectively.

### 2.2 Induction of EAN

The EAN group was immunized on day 2 and 7 p.i. by subcutaneous injection of P0 peptide 180-199 (GenScript, USA) mixed with *Mycobacterium tuberculosis* (BD, USA), emulsified saline, and Freund’s incomplete adjuvant (FIA; Sigma, USA). FIA plus *M. tuberculosis* is referred to as Freund’s complete adjuvant (FCA). Each mouse was given pertussis toxin (PTX; Millipore, USA) by tail intravenous injection on days 1, 3, and 5 p.i., respectively. The control group was managed in the same way as mentioned above but with phosphate-buffered saline (PBS) rather than P0 peptide.

### 2.3 The assessment of clinical severity and 50% Paw withdrawal threshold

The clinical severity of mice was assessed every day by two different examiners beginning on day 0 p.i. The severity for EAN was defined into 8 levels as follow: 0, normal; 1, flaccid tail; 2, ataxia or mild paraparesis; 3, moderate paraparesis; 4, severe paraparesis; 5, tetraparesis; 6, moribund; 7, death. Clinical severity for the intermediate level was set as 0.5. The “up–down” von Frey method was used to estimate the pain threshold, and monofilaments (Semmes-Weinstein monofilaments) with increasing force (g) from 0.07 to 4 g were used. Before the “up–down” method, the “ascending stimulus” method was taken to value the initial force that was near the 50% proportion responding and chose 1.4 g as our initial force [12].

### 2.4 Analysis of the proportions of macrophages in spleen mononuclear cells (MNCs) by flow cytometry

The mice were sacrificed at the attack and remission phase respectively. Single suspended macrophages were obtained from mice spleen, and centrifuged for 10 min. After red blood cell lysis, the splenocytes were harvested and adjusted the cell concentration to 1 × 10^6^/ml. The spleen MNCs were incubated with antibodies FITC-conjugated anti-mouse CD11b (BD Pharmingen™), APC-R700-conjugated anti-mouse F4/80 (BD Pharmingen™), PE-conjugated anti-mouse CD40 (BD Pharmingen™) for 30 min at 4°C in a dark environment. Then, cells were permeabilized with the fixation/permeabilization solution Kit (BD Pharmingen™) for 20 min. The intracellular molecules were stained with antibodies AF647-conjugated anti-mouse CD206 (BD Pharmingen™), eFlour450-conjugated anti-mouse arginase-1 (Arg-1) (Thermo), and PE-Cy7-conjugated anti-mouse inducible nitric oxide synthase (iNOS) (Thermo) for 30 min at 4°C in a dark environment. Flow cytometric analysis was performed by using FLOWJO 7.6.1 (Treestar). Both F4/80- and CD11b-positive were calculated as macrophages. In macrophages, both iNOS and CD40 positive were identified as pro-inflammatory macrophages, and either CD206 or Arg-1 positive was identified as anti-inflammatory macrophages.

### 2.5 Macrophages in DRG using immunohistochemical and immunofluorescence assays

The mice were anesthetized with 5% chloral hydrate and perfused with cold saline and 4% buffered formaldehyde. The lumbar DRG tissues were extracted and were cut lengthwise at a thickness of 8 µm and stored at 4°C in PBS containing 0.1% sodium azide. Macrophages were visualized using anti-F4/80 antibody (Servicebio, China) immunohistochemistry and immunofluorescence staining, respectively. The neurons and nerve fibers in DRG tissue are stained with anti-neurofilament heavy polypeptide antibodies (Servicebio, China). After blocking in PBS containing 30% BSA for 30 min at room temperature. Next, HRP-conjugated secondary antibody was added and incubated for 2 h at room temperature. Finally, the samples were fixed with PBS containing 10% DAPI for 2 min. The samples were observed using a confocal microscope (Zeiss LSM 800, Carl Zeiss Microimaging Inc., NY, USA) under a 20× objective. The percentage of the area of each group of sections positively expressed by macrophages was measured through ImageJ.

### 2.6 Statistical analysis

Statistical analysis was performed with Statistical Package for Social Sciences version 23.0 software (IBM, West Grove, PA, USA). The data was represented by mean ± standard and the means were compared using the rank sum test, and the Bonferroni–Dunn test for post hoc comparisons. For all statistical tests, *p* < 0.05 was considered to be significant.


**Ethical approval:** The research related to animals’ use has been complied with all the relevant national regulations and institutional policies for the care and use of animals. This study was approved by Ethics Committee of the Center for rehabilitation Medicine, Department of Neurology, Zhejiang Provincial People’s hospital.

## 3 Results

### 3.1 Clinical information of EAN

**Figure 1:**
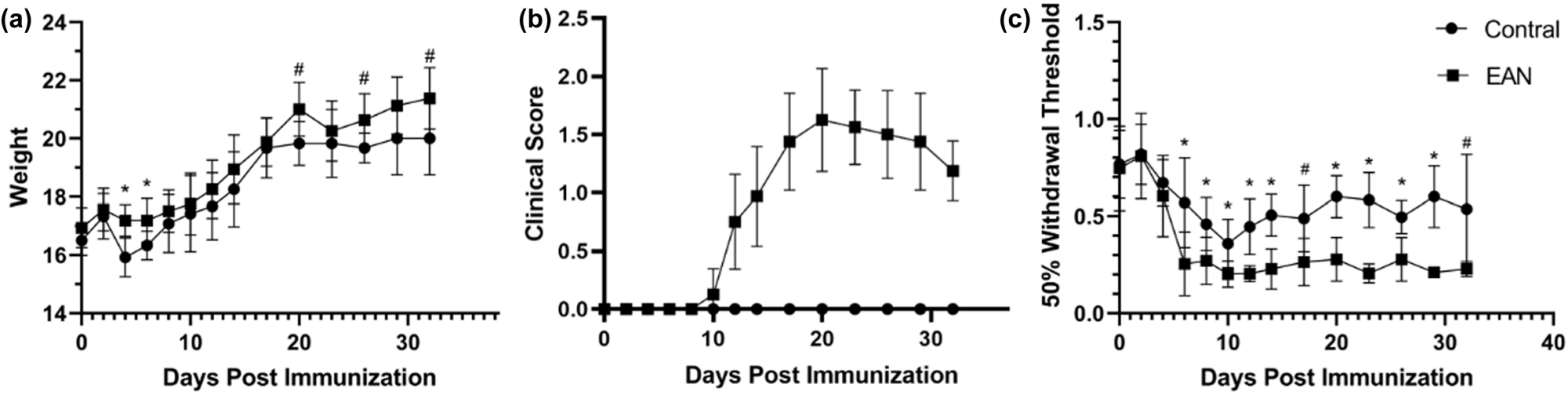
Clinical information in different time courses of the experimental autoimmune neuritis (EAN) and control groups. (a) The weight data of the EAN and control groups after immunization. (b) The clinical score of the two groups after immunization. (c) Comparison of 50% withdrawal threshold detection of the two groups. ^#^ *p* ＜ 0.05; *p ＜ 0.01.

The clinical course was compared between 12 mice with EAN induced by injecting P0 peptide 180-199 with FCA and the same number of control mice administered by PBS and FCA. After day 14 p.i., the clinical scores were compared between EAN and control group (*n* = 6, for each group). There was no significant difference in weight on day 0 p.i. In the early days (days 4–6 p.i.) and later days (days >20 p.i.), a tendency of higher weight was observed in the EAN group than in the control group (Figure 1a). Our data revealed that the symptoms of EAN mice started on day 10 p.i. and peaked on day 20 p.i., after which the clinical score showed a decreasing trend. (Figure 1b).

### 3.2 Lower pain threshold observed in the EAN mice

50% withdrawal threshold measuring, reflecting the pain threshold, starts on day 0 p.i. and was performed every two days. As exhibited in Figure 1c, both the EAN group and the control group showed a falling trend in pain thresholds and became stable after day 10 p.i. The pain thresholds were lower in the EAN group than in the control group and were statistically significant after day 6 p.i.

### 3.3 Macrophage infiltration in the DRG of EAN mice

**Figure 2:**
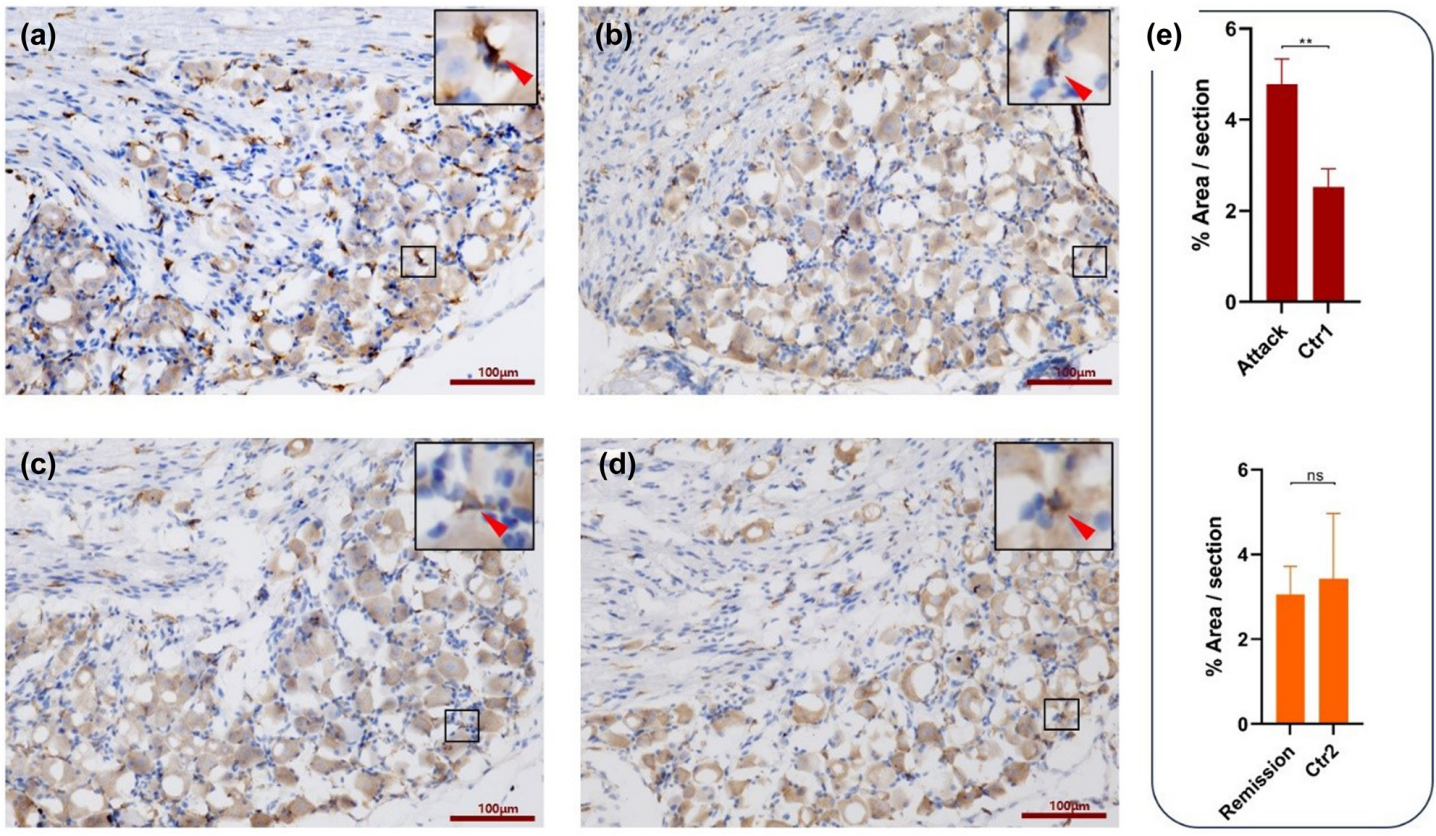
Immunohistochemical analysis of the four groups of DRG. Macrophages were stained by F4/80 antibodies and were shown as cells in the figures with brown membrane surface staining. The pictures with the black border are enlarged in the upper right corner, and macrophages are indicated with red arrows. (a) DRG section of the attack phase of mice with EAN. Abundant macrophages were observed around the neurons in the DRG of mice with EAN in the attack phase. (b) DRG section of the control group (Ctr1) for the attack phase. (c) DRG section of the remission phase of mice with EAN. (d) DRG section of the control group (Ctr2) for the remission phase. (e) Statistical plot for the percentage of the area of each group of sections positively expressed by macrophages. DRG, dorsal root ganglion; EAN, experiment autoimmune neuritis. **p ＜ 0.01; ns, *p* ＞ 0.05.

**Figure 3:**
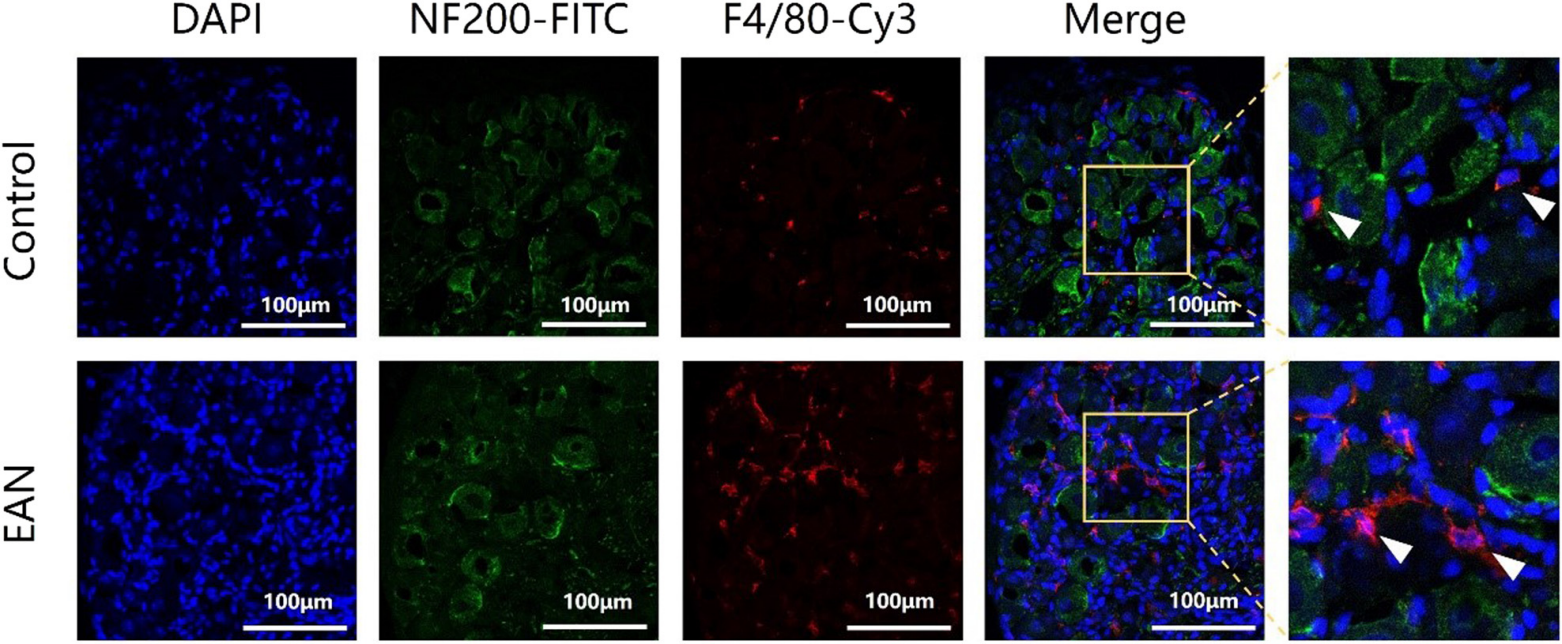
Immunofluorescence staining of the EAN and the control group in the attack phase was performed to verify the pathological changes in the attack phase. The pictures with the white border are enlarged in the upper right corner, and macrophages are indicated with white arrows Distribution of individual nuclei in DAPI blue fluorescent chromosomes now in DRG tissue; FITC green dye staining for intracellular NF200 shows the morphology of neurons and nerve fibers; Cy3 red dye stains the F4/80 membrane surface, reflecting macrophage morphology. Abundant macrophage infiltration was observed around neurons of the DRG of mice with EAN in the attack phase.

Immunohistochemistry staining with F4/80 antibody of lengthwise sections of DRG revealed that amounts of macrophages infiltrate the DRG tissue of EAN mice in the attack phase are significantly increased than remission phase as well as control group both in the attack and remission phases (*p* < 0.05, Figure 2a–e). In the remission phase, the amounts of macrophages infiltrate the DRG of the EAN group had no significant difference compared with the control group both in the attack and remission phases (*p* > 0.05). Immunofluorescence staining of the EAN and control groups in the attack phase was performed to verify the pathological changes in the attack phase, and as shown in Figure 3, abundant macrophage infiltration was observed in the DRG around the neurons of mice with EAN, consisting with the pathological result mentioned above.

### 3.4 The proportion of macrophages in EAN

**Figure 4:**
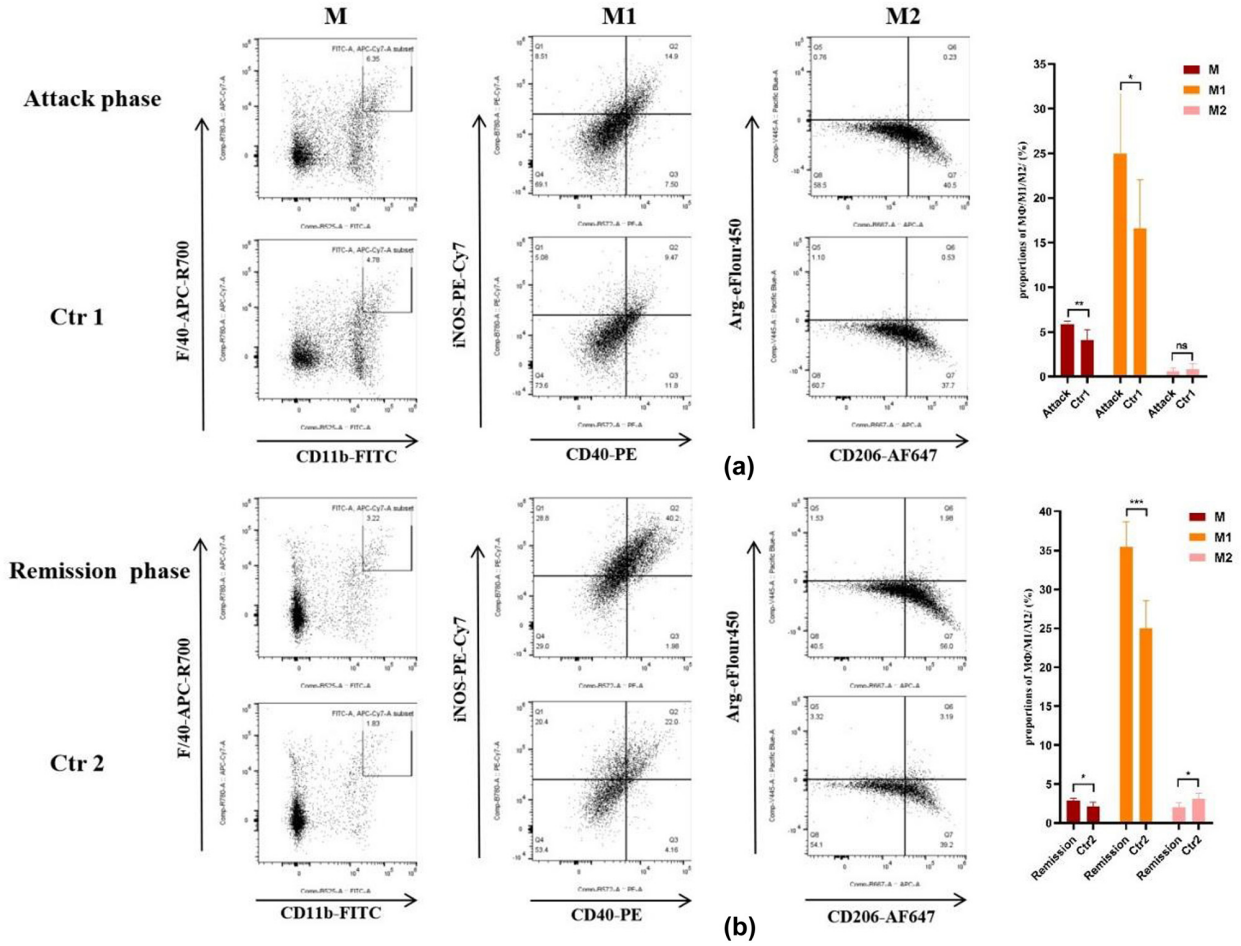
The proportions of macrophages in mice spleen MNCs were analyzed by flow cytometry. (a) The proportions of macrophages (CD11b + F4/80 + cells), pro-inflammatory macrophage (iNOS + CD40 + cells), anti-inflammatory macrophage (Arg + CD206 + cells) in mice spleen MNCs at attack phages; (b) the proportions of macrophages (CD11b + F4/80 + cells), pro-inflammatory macrophage (iNOS + CD40 + cells), and anti-inflammatory macrophage (Arg + CD206 + cells) in mice spleen MNCs at remission phages. M, macrophage; M1, pro-inflammatory macrophage; M2, anti-inflammatory macrophage. *p ＜ 0.05; **p ＜ 0.001; ***p ＜ 0.001;.

The proportion of macrophage, pro-inflammatory macrophage, and anti-inflammatory macrophage in mice spleen MNCs was analyzed by flow cytometric analysis in attack and remission phages. We found the proportions of macrophages (both CD11b and F4/80 positive) in EAN mice spleen MNCs were significantly higher than the control group (*p* < 0.01, Figure 4a) in the attack phase. The proportions of pro-inflammatory macrophages (both CD40 and iNOS positive) in macrophages of EAN mice spleen MNCs were increased significantly (*p* < 0.05, Figure 4a); however, the proportions of anti-inflammatory macrophages (both CD206 and Arg positive) had no significant difference compared with the control group (*p* > 0.05, Figure 4a) in the attack phase. In the remission phase, compared with the control group, we found the proportions of macrophages (both CD11b and F4/80 positive) in EAN mice spleen MNCs were higher (*p* < 0.05, Figure 4b). The proportions of pro-inflammatory macrophage (both CD40 and iNOS positive) in macrophages of EAN mice spleen MNCs were increased significantly (*p* < 0.001, Figure 4b), the proportions of anti-inflammatory macrophage (both CD206 and Arg positive) in macrophages of EAN mice spleen MNCs were decreased, while there was no statistical significance compared with the control group (*p* > 0.05, Figure 4b).

## 4 Discussion

In this study, we observed that EAN mice showed significantly lower pain threshold after 6 days p.i. We also found more macrophages infiltrated in the DRG of the EAN group in the attack phase and macrophages mostly surround neurons in DRG. In addition, the proportion of macrophage and pro-inflammatory macrophage in EAN mice spleen was significantly higher than the control group in the attack phase. The results in the present study suggested that in ENA mice the recovery course of clinical motor symptoms was earlier than the sensory. The infiltration of macrophage in the DRG may be associated with the falling of the pain threshold of EAN mice. Previous studies observed that neuropathic pain exists in mice with EAN [11,13]. Mechanical allodynia preceded the onset of motor deficits and remained after clinical signs disappeared [11]. Our results revealed that mice with EAN had stable neuropathic pain even when the clinical severity showed a decreasing trend, which is consistent with previous results and resembles the disease course of a large subset of GBS patients [14].

The precise mechanism of neuropathic pain in EAN remains unknown. However, recent studies have begun to focus on the role of macrophages in DRG, especially in their association with pathologic pain [3,4,15]. Previous reports found that the activated spinal microglia and astrocytes together with increased excitability in small DRG neurons were important for the initiation and maintenance of neuropathic pain in EAN [11,13]. Macrophage accumulation in the fourth lumbar DRG has been observed in Lumbar nerve injury, and the infiltrated macrophages cause neuropathic pain by deriving high mobility group box 1 (HMGB1) and upregulating Ca2^+^ channels in nociceptors [3]. Upregulation of micro ribonucleic acid-21 in DRG was found when the sciatic nerve was injured, which would promote pro-inflammatory macrophages, inhibit anti-inflammatory macrophages, and lead to neuropathic pain [15]. Chemotherapeutic agents, such as paclitaxel, directly stimulate macrophages and in turn secrete HMGB 1, leading to neuropathic pain [4]. In our study, we showed for the first time that macrophages significantly accumulated in the lumbar DRG of EAN mice in the attack phase. Our experiment also verified the existence of neuropathic pain in EAN mice. Based on the close association of macrophages in DRG and neuropathic pain by previous studies, our findings suggest that macrophage infiltration in DRG of EAN is associated with neuropathic pain.

Macrophages play important roles in EAN [6,8]. Pro-inflammatory macrophages secrete pro-inflammatory cytokines, contributing to disease development, while anti-inflammatory macrophages express anti-inflammatory molecules, promoting tissue repair and disease recovery [16]. Recent studies have found that promoting the polarization of macrophages from pro-inflammatory macrophages to anti-inflammatory macrophages in the spleen of EAN can promote recovery of EAN [7]. Our study also found a higher proportion of macrophages and pro-inflammatory macrophages in the spleen of mice with EAN, especially at the attack phase. Macrophages accumulating in DRG contain macrophages of circulating monocyte-derived infiltrating and of resident peripheral nerves [17]. Based on our study, we speculate that the proliferation of peripheral macrophages of mice with EAN may promote the infiltration and accumulation of macrophages into the DRG, which could contribute to the development of neuropathic pain. Further studies are needed to confirm our hypothesis.

Our study had several limitations. First, we did not perform functional experiments on macrophages so the mechanisms of macrophages in DRG-inducing neuropathic pain were still unclear. Further studies are needed to explore the mechanism. Second, we did not test the proportion of pro-inflammatory and anti-inflammatory macrophages in DRG. Further studies need to test the proportion of different types of macrophages in DRG using a suitable method.

## 5 Conclusion

The infiltration of macrophages in DRG may be associated with neuropathic pain of EAN and the presence of pro-inflammatory macrophages may be involved in hyperesthesia in EAN.
